# Production efficiency and nutritional potential of black soldier fly (*Hermetia illucens* Linnaeus, 1758) larvae with an evaluation of growth performance in Nile tilapia (*Oreochromis niloticus* Linnaeus, 1758)

**DOI:** 10.5455/javar.2026.m1059

**Published:** 2026-06-22

**Authors:** Fahima Israt, Md. Manzoorul Kibria, Mahmudul Hasan, Mohammad Shamsil Arafeen

**Affiliations:** 1 Department of Zoology, University of Chittagong, Chattogram-4331, Bangladesh; 2 Halda River Research Laboratory, University of Chittagong, Chattogram-4331, Bangladesh; 3 Integrated Development Foundation, Chattogram, Bangladesh

**Keywords:** Black soldier fly, Nile tilapia, proximate analysis, total larval yield, conversion rate, substrate reduction efficiency

## Abstract

**Objectives:** This study evaluated the production efficiency, nutritional value, growth performance, and economic viability of black soldier fly (BSF) (Diptera: Stratiomyidae) larvae as a sustainable alternative to commercial feed in Nile tilapia (*Oreochromis niloticus*) aquaculture.

**Materials and Methods:** BSF larvae were reared on a mixed substrate of four organic waste types. Monthly total larval yield, substrate reduction efficiency, and conversion rate were calculated to evaluate production efficiency. The nutritional composition of BSF larvae and commercial fish feed was determined through proximate analysis following the AOAC (1980) procedures. Growth performance was assessed using 30 Nile tilapia (15 fish per tank). Fish were fed daily at 5% of body weight. The control group received 100% commercial feed, whereas the experimental group received 100% BSF larvae.

**Results:** One-way ANOVA revealed significant (*p* < 0.05) monthly effects on total larval yield (1.44 × 10^–7^), substrate reduction efficiency (0.0002), and conversion rate (1.6 × 10^–8^). LSD post-hoc analysis identified April as the peak production month. Cohen’s *d* showed medium differences in dry matter (0.74–0.73) and moisture (0.75–0.76), very large differences in crude protein (3.26–4.08) and ash (3.11–3.32), and extremely large differences in ether extract (9.77–10.23), highlighting the superior nutritional value of BSF larvae. Weight gain did not differ significantly between feeding treatments (*p* > 0.05), although BSF-fed fish showed slightly greater growth. Cohen’s d revealed a medium effect size, suggesting a moderate advantage of BSF larvae.

**Conclusions:** Nile tilapia fed 100% BSF larvae achieved growth comparable to those fed commercial feed at less than half the feed cost (77.79 vs. 161 BDT/kg), demonstrating that BSF larvae are a nutritionally rich, economically viable, and sustainable protein source for aquaculture in Bangladesh.

## 1. Introduction

Bangladesh is one of the world’s leading fish-producing countries, with a total production of 4.915 million MT in FY 2022–2023. This remarkable achievement has enabled the country to attain self-sufficiency in fish production, ensuring an average daily dietary supply of 67.8 gm of fish per person. According to the FAO report The State of World Fisheries and Aquaculture 2022, Bangladesh ranked fourth in global tilapia production and third in Asia [1]. Among the cultured species, Nile tilapia (*Oreochromis niloticus*), one of the most widely farmed freshwater fishes, contributes significantly to global aquaculture, with production exceeding 6 million tonnes in 2020, dominated by Asia, followed by Africa and Latin America [2].

Global tilapia production faces multiple challenges, including rising feed costs, environmental factors, and disease outbreaks, as observed in countries such as China, Egypt, and Colombia, where feed alone accounts for 60–70% of total production costs, and increasing prices of fishmeal, soybean, and corn, coupled with supply chain disruptions, have further reduced profitability worldwide [3]. It is projected that by 2034, 84% of global fishmeal will be consumed by the aquaculture sector as feed, up from 78% in the base period [4]. In Bangladesh, financial limitations and high feed costs remain the most critical constraints for farmers, directly affecting productivity and profitability [5]. Feed cost makes up more than 52.4% of the production expense for intensive tilapia farms. Recent market data show commercial feed prices rising above Tk 73 per kg, up from around Tk 30–35 per kg in previous years, significantly squeezing farmers’ margins [6]. In Bangladesh, declining farm-gate tilapia prices alongside rising feed costs highlight the urgent need for cost-effective strategies, such as using locally available ingredients or feed innovations, to improve yields and maintain farmer profitability [7].

In this context, black soldier fly (BSF, *Hermetia illucens*) larvae are gaining global attention as a sustainable protein source [8]. BSF larvae convert organic waste into high-quality biomass rich in protein, lipids, and amino acids [9, 10]. Black soldier flies go through a complete metamorphosis life cycle, undergoing five main stages in its life cycle: egg, larval, last larval, pupal, and adult stages [11]. The major part of the black soldier fly (BSF) life cycle is indeed dominated by the larval and pupal stages, as these stages are the longest and most developmentally active phases. The larval stage is when most of the feeding and biomass accumulation occur, while the pupal stage is when metamorphosis happens. [12]. Females lay 500–900 eggs, which hatch within 4–5 days [13]. The larval stage lasts about 14–18 days [14], followed by a 7–10 day last larval period [11]. Pupation occurs within the hardened cuticle and lasts approximately 8 days [15]. The lifespan for adults is 5–8 days, during which they mate and do not feed [11].

Studies have shown that incorporating BSF larvae into aquafeeds can maintain or enhance growth performance, improve feed efficiency, and reduce reliance on conventional ingredients, while simultaneously addressing waste management challenges [16, 17, 18]. In Bangladesh, where urbanization generates approximately 25,000 tons of solid waste daily, BSF farming also offers an environmentally friendly solution to valorize organic waste [8, 11, 19].

Despite their potential, important knowledge gaps remain. Most existing studies have tested BSF larvae under controlled laboratory conditions or with single/binary waste substrates, providing limited insight into performance under real-world conditions. While several studies have evaluated the constraints of tilapia farming and the nutritional value of black soldier fly (BSF) larvae, there is still a lack of empirical evidence on the direct comparison of growth performance in Nile tilapia when fed BSF-based diets versus commercial feeds under practical farm conditions. This represents a key research gap, as most existing studies have been experimental or focused only on the proximate composition of BSF larvae without integrating farm-level growth trials, and economic analyses are also scarce, particularly in the South Asian context.

Therefore, this study addresses these gaps by: (i) evaluating larval yield, substrate reduction efficiency, and conversion rates of BSF larvae reared on a novel mixture of four organic wastes over several months; (ii) comparing the proximate nutritional composition of BSF larvae with commercial fish feed; and (iii) assessing the impact of 100% BSF substitution on growth performance parameters (percent weight gain, specific growth rate, feed conversion ratio, and feed efficiency) of Nile tilapia in a 50-day trial. This study is the first to evaluate BSF larvae production using a mixed organic waste substrate and assess its efficacy as a 100% replacement for commercial feed in Nile tilapia over a 50-day growth trial in Bangladesh. By integrating biological, nutritional, and economic assessments, this study provides novel insights into BSF larvae as a cost-effective and sustainable alternative to commercial feed in Bangladesh aquaculture.

Hypothesis: Based on the nutritional value of BSF larvae and their potential for waste valorization, we hypothesize that substituting commercial fish feed with 100% BSF larvae meal will sustain growth performance and feed efficiency of Nile tilapia while reducing feed costs.

## 2. Materials and Methods

### 2.1. Ethical approval

Ethical approval, Ref. No. AERB-FBSCU-20251029-(4), was obtained from the Animal Ethics Review Board, Faculty of Biological Sciences, University of Chittagong, Chattogram, Bangladesh. All experimental procedures involving fish handling were conducted in compliance with guidelines and regulations for the care and use of aquatic animals in research.

### 2.2. Study area

**Figure 1.**
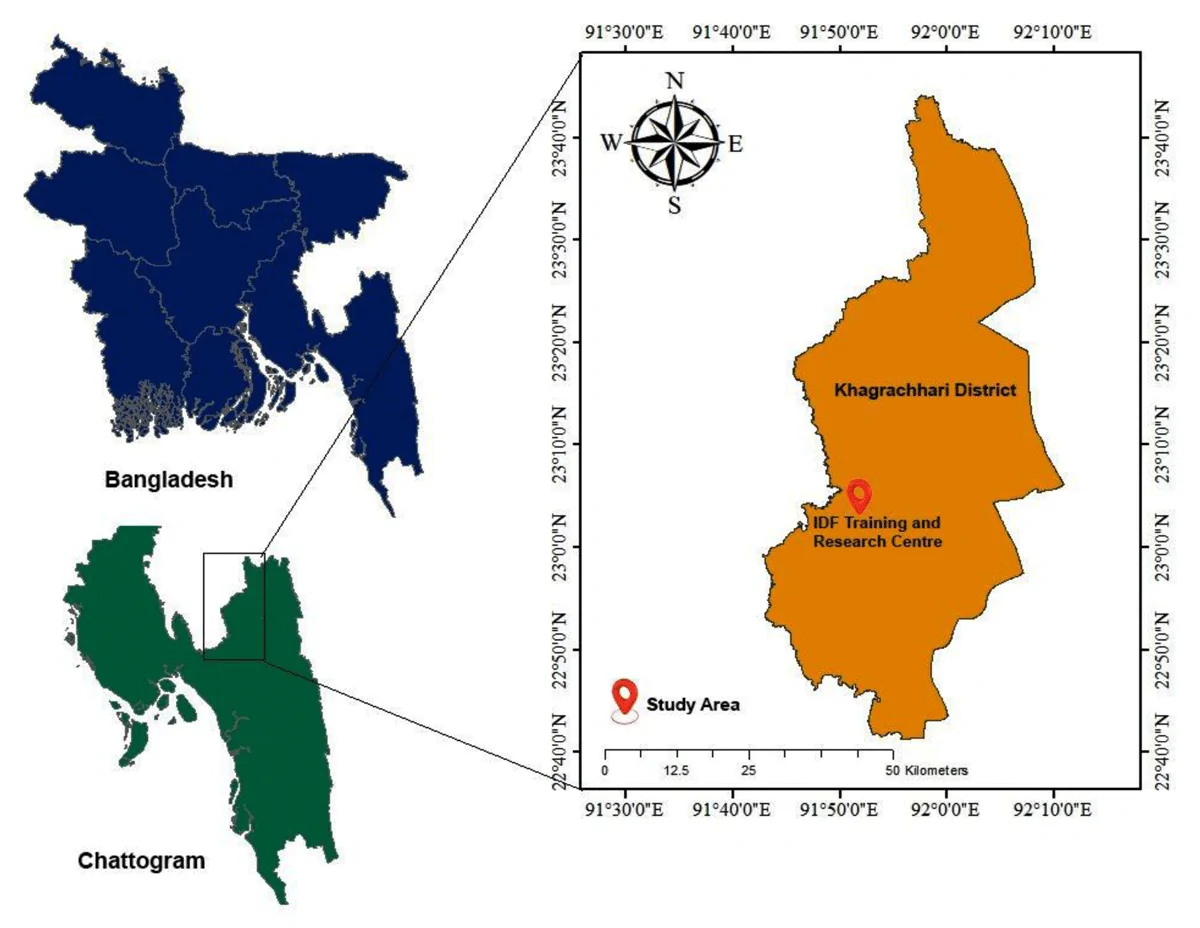
GIS Map of IDF Research and Training Center, Matiranga, Khagrachhari.

The BSF larval performance and feeding trials on Nile tilapia took place in the Integrated Development Foundation (IDF) research and training center, Matiranga, Khagrachhari (22°56ʹ–23°24ʹN, 91°44ʹ–91°58ʹE), Chattogram, Bangladesh (Figure 1), where environmental temperatures vary from 6.7°C in January to 42.2°C in May, and winter rainfall averages 87 mm. Proximate analysis was carried out at the Department of Nutrition and Animal Science, Chattogram Veterinary and Animal Science University (22.3639°N, 91.8040°E), Chattogram, Bangladesh.

### 2.3. BSF larval performance experiment

#### 2.3.1. Source of larvae

BSF larvae were obtained from a local supplier in Sitakunda, Bangladesh, and maintained at the IDF BSF unit, Khagrachhari. Adults were kept in a mesh enclosure (1 mm mesh size) under ambient conditions (27–32°C; 60–80% RH). A sugar solution was sprayed on potted plants to extend adult life and promote oviposition.

#### 2.3.2. Substrate preparation

Organic wastes were collected from Matiranga Bazar, consisting of poultry litter, vegetable waste, animal by-products (chicken giblets and fish scraps), and cow dung. The collected waste was sorted to remove contaminants, ground with a crusher, and homogenized in a fixed weight ratio of 4:3:2:1 (w/w) for poultry litter, vegetable waste, animal by-products, and cow dung, respectively.

#### 2.3.3. Egg collection and hatching

Oviposition was stimulated using buckets containing decomposing poultry and fish scraps, with stacked wooden blocks provided as egg-laying substrates. Eggs were collected daily, weighed, and transferred to nursery trays containing moist chick mash (~60% moisture). Neonates were maintained on chick mash for five days before transfer to experimental containers.

#### 2.3.4. Larval rearing and harvesting

Uniform larvae (5 gm per replicate) were introduced into ten plastic bowls (18 L capacity), each containing 10 kg of the mixed substrate. Larvae were reared under ambient conditions until reaching the last larval stage, at approximately 13–22 days, indicated by a color change from white to brown. Larvae were separated from residual substrate using a 3–5 mm mesh sieve and weighed. Approximately 80% of larvae were harvested for analysis, while 20% were retained to sustain the colony.

#### 2.3.5. Post-harvest processing

Harvested larvae were washed, blanched in boiling water for 5 min, and sun-dried. Dried larvae were stored in airtight containers for both use as feed and for proximate analysis.

### 2.4. Nutritional evaluation analysis

Proximate analysis is widely used for the nutritional evaluation of feed across the world. It partitions feed into six major components: moisture (or dry matter), crude protein (CP), crude fiber (CF), ether extract (EE), total ash (TA), and nitrogen-free extract (NFE). Among these, NFE is calculated indirectly. This systematic partitioning provides a comprehensive understanding of the feed’s nutritive value. This experiment was performed on dried BSF larvae and on commercial fish feed (starter and grower) to compare moisture, crude protein, crude fat, and ash content following AOAC protocols [20]. Moisture content is measured based on the oven-drying method at 105°C to constant weight. Ash content was calculated by burning the samples in a furnace at a temperature of 550°C. Crude protein content analysis was carried out based on the Kjeldahl method. Crude fat content was measured by Soxhlet extraction with petroleum ether. crude fiber (CF) and nitrogen-free extract (NFE) were not determined in this study.

### 2.5. Feeding trials on Nile tilapia

#### 2.5.1. Experimental design

A total of 30 Nile tilapia (*O. niloticus*) juveniles (53 ± 1.84 gm) were used for the feeding trial conducted at IDF Farm, Matiranga, Khagrachhari (February–March 2025), Bangladesh. Fish were acclimatized on a commercial starter diet prior to the trial. Following acclimation, fish were randomly allocated into two experimental groups (15 fish/tank; two tanks in total, one per treatment) at a stocking density of 15 fish per 0.949 m^3^. Each tank was housed in an identical experimental unit (Length = 1.78 m, Width = 2.44 m, Depth = 0.78 m, Edge height = 0.25 m).

The two dietary treatments were as follows:

- T_1_ (Treatment): 100% black soldier fly (BSF) larvae diet.
- T_2_ (Control): 100% commercial fish feed (starter and grower pellets).

Fish were hand-fed twice daily (08:00–09:00 and 15:00–16:00 h) at 5% of their body weight, with rations adjusted every 10 days based on group biomass. The feeding trial lasted for 50 days.

#### 2.5.2. Water management and tank maintenance

The feeding trial was conducted under captive conditions in tanks at IDF Farm, Matiranga, Khagrachhari. Water was sourced from an underground well and was partially renewed every 3 days to maintain a stable environment. Tanks were thoroughly cleaned prior to stocking and visually monitored daily to ensure clarity and the absence of foul odor. No direct measurements of temperature, pH, dissolved oxygen, or ammonia were performed.

#### 2.5.3. Feed cost calculation description

The feed cost was calculated based on the actual feed intake per fish during the 50-day experimental period. Fish in each tank were fed daily at 5% of their body weight, adjusted every 10 days according to mean body weight measurements. The total feed supplied per fish over the entire trial was multiplied by the unit cost of each diet. The feed cost per kilogram of fish biomass gain was then derived by dividing the total feed expenditure by the net weight gain of fish.

### 2.6. Data collection and research variables

During harvest events, total larval yield, substrate reduction efficiency, and conversion rate were recorded in each experimental month. Dried BSF larvae sample and commercial fish feed (starter and grower) were carried out to evaluate nutritional composition. During feeding trials on Nile tilapia, conducted for 50 days, data were collected at 10-day intervals. At each sampling point, percent weight gain (PWG), specific growth rate (SGR), feed conversion ratio (FCR), feed conversion efficiency (FCE), and feed cost per fish were determined (Table 1).

**Table 1.** Parameters and formula used in the study.

Parameter	Formula/Description
Total larval yield	Monthly values calculated as the mean of all harvests conducted during that month
Substrate Reduction Efficiency (SRE)	(Total waste added - Residue waste after treatment) / Total waste added
Conversion Rate (CR)	(Total larval mass / Total waste added) × 100
Percent Weight Gain (PWG)	(Mean final weight – Mean initial weight) / Mean initial weight × 100
Specific Growth Rate (SGR)	(logW_2_ – logW_1_) / Time × 100
Feed Conversion Ratio (FCR)	Weight of the feed offered / Weight Gain
Feed Conversion Efficiency (FCE)	1/ FCR
Feed Cost Per Fish	Daily feed (gm) × Number of days × Feed cost per (gm)

### 2.7. Data and statistical analyses

The data were presented as the mean ± standard deviation of the mean. A one-way analysis of variance was performed for the total larval yield, substrate reduction efficiency, and conversion rate to compare the means of the different variables at a 5% level of significance. Again, an LSD post hoc test was applied based on the larval yield to identify which month is significantly different in larval production. Cohen’s d test was used to assess effect size on the nutritional profiles of both diets. A one-way ANOVA was conducted on growth performance parameters, followed by Cohen’s d test. For these analyses, Microsoft Excel and SPSS Statistics software were used.

## 3. Results and Discussion

### 3.1. Performance of BSF larvae

Total larval yield, substrate reduction efficiency, and conversion rate of *Hermetia illucens* showed significant variation (*p* < 0.05) throughout the experimental period (September 2024 to June 2025). All these three metrics were recorded highest in April, with an average of 19.53 ± 0.096 kg, 47.63 ± 0.15%, and 19.60 ± 0.10%, while the lowest were observed in January: 16.76 ± 0.177 kg, 40.95 ± 0.35%, and 16.70 ± 0.14% (Table 2). LSD post hoc analysis on larval yield revealed that April is the peak month, which was significantly higher than all other months. During the study period, it was observed that the temperature in April was around 32°C (± 0.58) and the relative humidity was approximately 73% (73 ± 5.57)%, which aligns with the optimal rearing conditions reported by a previous study [21].

**Table 2.** Total larval yield, substrate reduction efficiency, and conversion rate of BSF larvae across different months (September 2024 – June 2025).

Month	Total larval yield, (kg)	Substrate reduction efficiency, %	Conversion rate, %
September, 24	17.31 ± 0.085^abc^	42.50 ± 1.27^ab^	17.25 ± 0.07^abc^
October, 24	17.39 ± 0.046^abc^	43.17 ± 0.32^bc^	17.36 ± 0.05^bcd^
November, 24	17.50 ± 0.021^bc^	44.30 ± 2.10^cd^	17.45 ± 0.07^cd^
December, 24	17.33 ± 0.110^abc^	42.75 ± 1.63^ab^	17.30 ± 0.10^bcd^
January, 25	16.76 ± 0.177^a^	40.95 ± 0.35^a^	16.70 ± 0.14^a^
February, 25	16.83 ± 0.092^a^	41.75 ±0.07^a^	16.80 ± 0.14^a^
March, 25	17.73 ± 0.444^bcd^	45.93 ± 2.42^cd^	17.70 ± 0.45^ce^
April, 25	19.53 ± 0.096^e^	47.63 ± 0.15^e^	19.60 ± 0.10^f^
May, 25	18.04 ± 0.546^cd^	45.27 ± 1.27^cd^	18.00 ± 0.51^e^
June, 25	17.06 ± 0.318^ab^	42.80 ± 0.60^bc^	17.03 ± 0.32^ab^

Note: Values are expressed as mean ± standard deviation. Different superscript letters (a–e) within the same column indicate a significant difference (*p* < 0.05). Columns that share the same letter are not significantly different. For the parameters measured, the *p*-values were as follows: total larval yield (1.44 × 10^–7^ < 0.05), substrate reduction efficiency (0.0002 < 0.05), and conversion rate (1.6 × 10^–8^ < 0.05).

Seasonal variations in temperature and relative humidity throughout the year may influence larval production by affecting the biological and physiological processes of the larvae, as documented in previous studies [22, 23]. This monthly production data provided critical insights for the commercial adoption of BSF farming, as identifying peak and low-yield months can guide operational planning. Addressing environmental and operational constraints during low-yield months could enhance overall efficiency, supporting stable and scalable BSF production under variable climatic conditions.

### 3.2. Nutritional analysis of BSF larvae and commercial fish feed

The proximate composition of BSF larvae showed higher mean crude protein (43.23 ± 2.24), mean ash content (18.48 ± 2.40), and mean ether extract (21.15 ± 0.81) than commercial fish feed in three different months (January, March, and May), as shown in Table 3. To represent the magnitude and practical relevance of the nutrient differences, Cohen’s d effect size measurement was done as BSF vs. commercial starter and BSF vs. commercial grower feed. The result revealed medium differences in dry matter (0.74–0.73) and moisture (0.75–0.76), markedly higher differences in crude protein (3.26–4.08) and ash (3.11–3.32), and extremely large differences in ether extract (9.77–10.23), confirming the superior nutritional value of BSF larvae over commercial feeds (starter & grower).

**Table 3.** Comparison of nutritional analysis between black soldier fly larvae and commercial feed (starter and grower).

Serial No.	Name of Nutrient	Result Percentage (%)		
		BSF larvae	Commercial fish feed	
			Starter	Grower
1	Dry matter (DM)	88.94 ± 4.75	92.51	92.58
2	Moisture	11.02 ± 4.70	7.49	7.42
3	Crude protein (CP)	43.23 ± 2.24	28.78	24.85
4	Ash	18.48 ± 2.40	10.63	10.25
5	Ether Extract/Fat	21.15 ± 0.81	5.62	5.26

As previously studied, insects exhibit distinct metabolic pathways compared to conventional animals. They are ectothermic and do not expend energy on thermoregulation. Consequently, the amount and quality of protein in the diet directly influence larval development [24, 25]. This highlighted the rich protein and fat content of BSF larvae, making them a valuable feed ingredient not only for poultry but also potentially for fish diets, where it may enhance growth performance [26].

Based on another review, BSF reared on animal manure (cattle, chicken, and pig) can reach ≥ 40% protein and ≥ 20% fat, indicating that substrate type strongly influences larval nutrition [27]. This underscores the importance of optimizing harvest time to ensure maximum nutritional output, especially when protein content is a target parameter. This highlighted the importance of BSF larvae as a nutrient-rich feed ingredient, providing high protein and fat content that can enhance the nutritional quality of aquafeeds.

### 3.3. Feeding trials’ effect on fish growth

Growth performance metrics favored the BSF diet, showing higher percent weight gain (11.09 ± 4.20%), specific growth rate (0.45 ± 0.15%), and feed conversion efficiency (14.74 ± 5.51%). In contrast, the commercial diet showed a higher feed conversion ratio (7.45 ± 2.68) (Table 4). One-way ANOVA indicated no statistically significant differences (*p* > 0.05) between treatments: PWG (F = 2.54, *p* = 0.1426), SGR (F = 2.53, *p* = 0.1432), FCR (F = 0.23, *p* = 0.6439), and FCE (F = 0.49, *p* = 0.4965). Even though the BSF-fed group showed slightly higher growth than the commercial feed group.

**Table 4.** Comparison of percent weight gain (PWG), specific growth rate (SGR), feed conversion ratio (FCR), and feed conversion efficiency (FCE) of Nile tilapia fishes fed on black soldier fly and commercial feed (mean ± SD).

Feeding group	Parameters			
	PWG %	SGR %	FCR	FCE %
BSF larvae	11.09 ± 4.20^a^	0.45 ± 0.15^a^	7.45 ± 2.68^a^	14.74 ± 5.51^a^
Commercial fish feed	9.10 ± 2.16^a^	0.37 ± 0.08^a^	8.7 ± 2.51^a^	12.13 ± 2.57^a^

Note: Values are expressed as mean ± standard deviation. The superscript letter ‘a’ in the table represents that there is no significant difference (*p* > 0.05) within the same column. Columns sharing the same letter are not significantly different.

Table 5 represents effect size analysis (Cohen’s d) confirmed moderate differences between diets, with medium effects observed for percent weight gain (*d* = 0.59), specific growth rate (*d* = 0.63), feed conversion ratio (*d* = –0.48), and feed conversion efficiency (*d* = 0.59), suggesting practical relevance of these differences despite the lack of statistical significance. The slightly higher growth observed in BSF-fed fish may be attributed to the high-quality protein and lipid content of the larvae, which provide essential amino acids and energy for growth and metabolism. Moreover, BSF contains chitin, which may stimulate gut microbiota and enhance nutrient absorption, potentially improving feed utilization efficiency.

**Table 5.** Effect size comparison between Black soldier fly larvae and commercial feed in Nile tilapia.

Parameter	BSF Larvae (*n* = 5)	Mean ± SD	Commercial Feed (*n* = 5)	Mean ± SD	Pooled SD	Cohen’s d	Effect Size
PWG	17.42, 10.16, 12.88, 7, 8	11.09 ± 4.20	9.77, 9.58, 11.71, 8.67, 5.79	9.10 ± 2.16	3.46	0.59	Medium
SGR	0.69, 0.42, 0.52, 0.29, 0.35	0.45 ± 0.15	0.40, 0.39, 0.48, 0.36, 0.24	0.37 ± 0.08	0.127	0.63	Medium
FCR	4, 7.38, 5.82, 10.7, 9.37	7.45 ± 2.68	7.69, 7.82, 6.4, 8.65, 12.95	8.7 ± 2.51	2.54	–0.48	Small
FCE	23, 13.54, 17.17, 9.33, 10.66	14.74 ± 5.51	13, 12.78, 15.62, 11.56, 7.72	12.13 ± 2.57	4.37	0.59	Medium

These findings align with previous research in tilapia [28], rainbow trout [29], and carp [30], which demonstrated comparable or improved growth when fishmeal was partially or fully replaced with BSF larvae. Differences in observed growth among studies may be influenced by larval substrate, inclusion level, and feeding duration. The results of this study indicate that BSF larvae can be used as an alternative protein source for Nile tilapia, supporting growth performance comparable to or slightly higher than commercial feed. These findings are consistent with previous studies that reported that substituting fish meal with BSF larvae in aquafeeds can support growth and overall health in aquaculture species [31].

Beyond growth performance, feed cost is another critical factor influencing the feasibility of BSF inclusion, and this was also evaluated in the present study. At the end of the 50-day trial, the feed cost per kilogram of fish production was 161 BDT for the commercial diet and 77.79 BDT for the BSF larvae diet. This represents a cost reduction of more than 50% when using BSF larvae as the sole protein source compared to commercial feed.

## 4. Conclusions

The study provides valuable insights in larval production across different months, identifying the periods of highest and lowest production, which will help in adopting targeted strategies to address environmental and operational challenges. Combining multiple waste streams not only enhances the efficiency of waste management but also demonstrates a more practical and scalable model. Mixed substrates support effective larval growth and produce nutritionally valuable biomass. With higher growth performance parameters, BSFL meal can be used as a sustainable, cheaply affordable, promising substitute in aquaculture over commercial fish feed. This study confirms that black soldier fly larvae are capable of efficiently converting low-nutrient food into high-value biomass, demonstrating excellence in both waste reduction and nutrient conversion.

## Data Availability

The data presented in this study are available from the corresponding author upon reasonable request.

## List of abbreviations

BSF, Black soldier fly; IDF, Integrated Development Foundation; AOAC, Association of Official Analytical Chemists; PWG, percent weight gain; SGR, specific growth rate; FCR, feed conversion ratio; FCE, feed conversion efficiency.
